# Isolated palpitations and ventricular pre‐excitation

**DOI:** 10.1002/joa3.12358

**Published:** 2020-05-13

**Authors:** Miguel A. Arias, Marta Pachón, Cristina Martín‐Sierra

**Affiliations:** ^1^ Arrhythmia Unit Department of Cardiology Complejo Hospitalario Universitario de Toledo Toledo Spain

**Keywords:** accessory pathway, electrophysiology, tachycardia

## Abstract

A 27‐year‐old male was referred for further assessment after being evaluated by his general practitioner for isolated palpitations. A twelve‐lead electrocardiogram was performed in which sinus rhythm with ventricular pre‐excitation were observed. Electrophysiologic study demonstrated the presence of a fasciculoventricular accessory pathway.

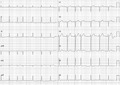

## 1 ECG CHALLENGE

A 27‐year‐old male patient was referred to our Unit for further assessment after being evaluated by his general practitioner for isolated palpitations. The symptoms were not related to any situation and were perceived as skipped beats. A 12‐lead electrocardiogram was performed in which sinus rhythm with ventricular pre‐excitation was observed. The patient denied having rapid palpitations. Transthoracic echocardiography ruled out the presence of any form of significant structural heart disease and physical examination was unremarkable. What is the most likely location of the accessory pathway? How should the patient be managed?

## 2 RESPONSE TO ECG CHALLENGE

**FIGURE 1:**
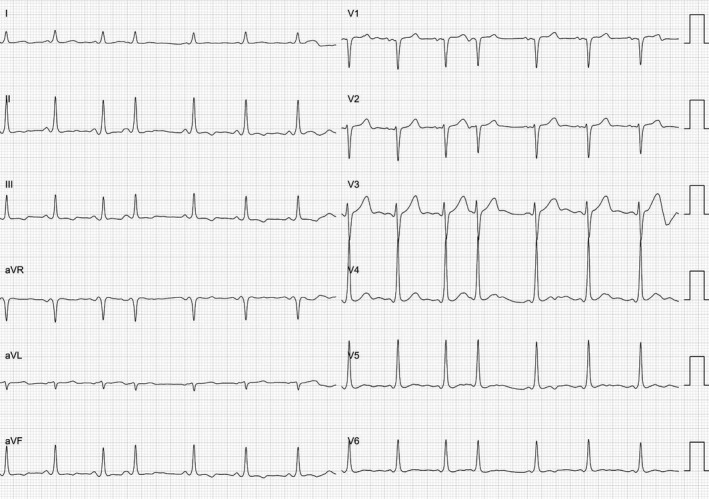
Twelve‐lead electrocardiogram of the patient. The trace shows normal sinus rhythm at a rate of 90 beats/min with a PR interval of 120 ms and small delta wave positive in inferior leads and negative in precordial lead V1. An atrial premature complex conducted to the ventricle is also evident (fourth beat)

On Figure 1, minimal pre‐excitation with positive delta wave in inferior leads, V5, and V6, and negative in V1, raised our suspicion for the existence of a fasciculoventricular accessory pathway (FVAP), although the existence of an anteroseptal atrioventricular accessory pathway (AP) could not be ruled out. FVAPs represent a very uncommon form of pre‐excitation resulting from an atypical AP connecting either the His bundle or the fascicles and the ventricular myocardium and the anteroseptal region.^1^ For that reason, the earliest ventricular activation occurs at the His bundle region. In patients with FVAPs without associated structural heart disease, pre‐excitation is minimal and always present in normal sinus rhythm. The pattern of pre‐excitation appears with an anteroseptal location. However, when compared to electrocardiogram from patients with anteroseptal APs, electrocardiogram of patients with FVAPs was found to have significantly lower delta wave amplitudes, longer PR intervals, and narrower QRS durations.^2^

**FIGURE 2:**
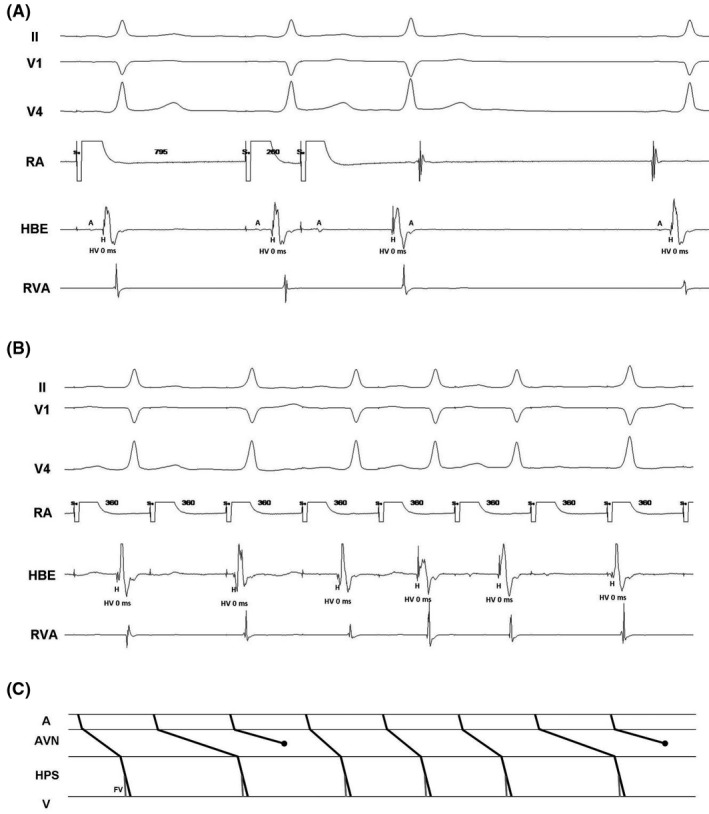
A, Electrocardiographic leads II, V1, and V4 and intracardiac recording from right atrium, His bundle, and right ventricular apex during atrial extrastimulus testing. B, Electrocardiographic leads II, V1, and V4 and intracardiac recording from right atrium, His bundle, and right ventricular apex during rapid atrial pacing. C, Ladder diagram explaining the phenomena observed in panel (B). Panel (A) shows the end of an atrial pacing train at 795 ms followed by the introduction of a single atrial extrastimulus coupled at 260 ms During the train, ventricular pre‐excitation is observed, with an HV interval of 0 ms (normal between 35 and 55 ms), and after the atrial extrastimulus, conduction through the atrioventricular node is prolonged very significantly (AH interval), pre‐excitation continues to the same degree, and a single atrioventricular nodal echo beat occurs as well. For the last beat shown in the figure, which is a sinus beat, the degree of pre‐excitation is also the same (HV interval of 0 ms). In panel (B), during a 360 ms atrial pacing train, Mobitz I‐type second‐degree atrioventricular block is observed due to block in the atrioventricular node, with persistence of the same degree of pre‐excitation in all conducted beats regardless of PR interval length. In panel (C), the presence of the fasciculoventricular pathway inserted to the His‐Purkinje system distally to the His bundle is represented, which allows the ventricle to be slightly pre‐excited for the conducted beats. A, atrium; AVN, atrioventricular node; FV, fasciculoventricular accessory pathway; H, His signal; HBE, His bundle electrogram; HPS, His‐Purkinje system; HV, His‐to‐ventricle interval; RA, right atrium; RVA, right ventricular apex; V, ventricle

The gold standard for definite diagnosis is the electrophysiologic study. Indeed, many patients with FVAPs have additional arrhythmic substrates, including additional typical rapidly conducting APs and atrioventricular nodal re‐entry that can be treated by catheter ablation.^2^ An electrophysiological study was performed in our patient. Baseline pre‐excitation was confirmed (HV interval: 0 ms) with earliest ventricular activation at His bundle area (Figure 2). Ventriculoatrial conduction was concentric and decremental through the normal conduction system. Dual atrioventricular nodal physiology and single atrioventricular nodal echo beats were demonstrated (Figure 2) with no induction of any type of specific tachycardia. Several observed findings confirmed the presence of an FVAP. Paced premature atrial beats (Figure 2) produced physiological prolongation of the PR and AH intervals but with a fixed degree of pre‐excitation (confirmed invasively by a constant and positive short HV interval). Moreover, induced atrioventricular Wenckebach block by rapid atrial pacing associated with a fixed degree of pre‐excitation and a constant, short HV interval was demonstrated (Figure 2, panel B). In patients with typical APs, the same maneuver leads to an increase in the degree of pre‐excitation. As expected, loss of atrioventricular conduction was associated with loss of pre‐excitation (Figure 2). No treatment was applied due to it is well known that FVAPs are never been actively involved in any type of supraventricular tachycardia. Spontaneous occasional atrial premature beats were responsible for patient's symptoms. The presence of FVAPs also does not increase the risk of sudden death with rapid antegrade conduction from pre‐excited atrial fibrillation because they are infranodal structures. However, in spite of the absence of clinical implications, their precise diagnosis is very important to distinguish them from anteroseptal APs, since a misdiagnosis could lead to trying to eliminate them with ablation with the potential risk of damaging the normal conduction system due to its proximity.

Although most cases of FVAPs are observed in patients without structural heart disease, these atypical APs have also been associated with various genetic syndromes. Among them, the rare glycogen storage cardiomyopathy, a genetic disorder caused by PRKAG2 gene mutation with a high incidence of complete atrioventricular block and atrial arrhythmias.^3^ Clinicians should be aware of this rare possibility.

## CONFLICT OF INTEREST

None.
